# LncRNA HCG18 affects aortic dissection through the miR-103a-3p/HMGA2 axis by modulating proliferation and apoptosis of vascular smoothing muscle cells

**DOI:** 10.1016/j.clinsp.2024.100400

**Published:** 2024-07-31

**Authors:** ZhiHong Yang, YuanSheng Cui, ShuGuo Xu, LongBiao Li

**Affiliations:** Department of Invasive Technology, Ningde Municipal Hospital of Ningde Normal University, Ningde City, Fujian Province, China

**Keywords:** LncRNA HCG18, miR-103a-3p, HMGA2, VSMCs, Aortic dissection

## Abstract

**Background:**

Aortic Dissection (AD) is a vascular disease with a high mortality rate and limited treatment strategies. The current research analyzed the function and regulatory mechanism of lncRNA HCG18 in AD.

**Methods:**

HCG18, miR-103a-3p, and HMGA2 levels in the aortic tissue of AD patients were examined by RT-qPCR. After transfection with relevant plasmids, the proliferation of rat aortic Vascular Smoothing Muscle Cells (VSMCs) was detected by CCK-8 and colony formation assay, Bcl-2 and Bax was measured by Western blot, and apoptosis was checked by flow cytometry. Then, the targeting relationship between miR-103a-3p and HCG18 or HMGA2 was verified by bioinformation website analysis and dual luciferase reporter assay. Finally, the effect of HCG18 was verified in an AD rat model induced by β-aminopropionitrile.

**Results:**

HCG18 and HMGA2 were upregulated and miR-103a-3p was downregulated in the aortic tissues of AD patients. Downregulating HCG18 or upregulating miR-103a-3p enhanced the proliferation of VSMCs and limited cell apoptosis. HCG18 promoted HMGA2 expression by competing with miR-103a-3p and restoring HMGA2 could impair the effect of HCG18 downregulation or miR-103a-3p upregulation in mediating the proliferation and apoptosis of VSMCs. In addition, down-regulation of HCG18 could improve the pathological injury of the aorta in AD rats.

**Conclusion:**

HCG18 reduces proliferation and induces apoptosis of VSMCs through the miR-103a-3p/HMGA2 axis, thus aggravating AD.

## Highlights

• Downregulating HCG18 enhances VSMC proliferation and reduces apoptosis.
• HCG18/miR-103a-3p/HMGA2 axis affects VSMC proliferation and apoptosis.
• Downregulating HCG18 improves the pathological injury of the aorta in AD rats.

## Graphical abstract

**Figure:**
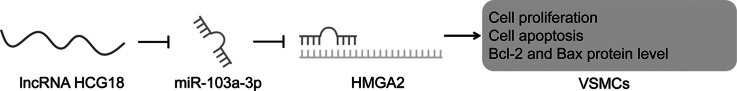

## Introduction

Aortic dissection, an acute macrovascular disease with a dangerous condition and a high mortality rate, can be divided into Stanford type A and Stanford type B depending on whether the dissection affects the ascending aorta.^1^, ^2^, ^3^, ^4^ Acute Stanford Type A Aortic Dissection (TAAD) has the characteristics of sudden onset, rapid progression, and high mortality.^5^ In recent years, the use of advanced imaging technology has made TAAD clear diagnosis is no longer difficult, but there are still many questions about its pathogenesis and treatment options. Studies found that AD is a comprehensive pathological change process caused by pathological changes involving multiple blood vessel constituents, such as human aortic Smooth Muscle Cells (VSMCs) and extracellular matrix.^6^^,^^7^ Surgical operation is the only approach for curing TAAD; however, the surgery is invasive and costly, and mortality rates are still high, even after treatment. Therefore, understanding the pathogenesis of AD and searching for biomarkers may reveal new therapeutic strategies to reduce clinical mortality.

Long non-coding RNAs (lncRNAs) are a category of non-coding transcripts with longer than 200 nucleotides, which have attracted more and more attention of researchers. LncRNAs are closely related to cardiovascular diseases, cancer, and neurodegenerative diseases.^8^, ^9^, ^10^ A member of the HLA complex, lncRNA HCG18 presents an abnormal expression profile in gastric cancer,^11^ melanoma,^12^ osteosarcoma,^13^ and nasopharyngeal carcinoma.^14^ HCG18 is overexpressed in intervertebral disc degeneration and it can promote the degradation of extracellular matrix in nucleus pulposus cells.^15^ Additionally, HCG18 inhibits proliferation and induces apoptosis of VSMCs,^16^ indicating that HCG18 may be related to AD. However, the regulatory mechanism of HCG18 in AD progression remains unclear.

MicroRNAs (miRNAs) are a family of small non-coding RNAs with a length of 18‒22 nucleotides. Evidence suggests that during normal physiological and pathological conditions, lncRNAs act as competing endogenous RNAs that competitively bind and sequester their target miRNAs, thereby counteracting miRNA-mediated repression of targeted mRNAs, indirectly regulating the expression of the miRNA target genes.^17^^,^^18^ In addition, in this study, the authors found that there are target binding sites with HCG18 with miR-103a-3p and miR-103a-3p with HMGA2. Therefore, the aim of this study was to investigate the role and potential mechanism of HCG18/miR-103a-3p/HMGA2 axis in the development of AD and to provide new targets for targeted therapy of AD.

## Methods

### Surgical indications

Patients presenting with signs and symptoms of TAAD were always treated on an emergency basis as soon as the diagnosis was confirmed by transthoracic 2D echo and/or angio-computed tomography. Potential contraindications were progressively reduced, current indications including patients with systemic malperfusion, with irreversible neurological damage, and advanced age. The surgical strategy was mainly dictated by the preoperative imaging and the intraoperative findings. Graft replacement of the ascending aorta, always extended to include the hemiarch, was considered as a limited repair, performed when the entry tear was found only in the ascending aorta; dilatation of the aortic arch or presence of arch tears, evidenced by routinely performing an open distal anastomosis, represented an indication to replace both the ascending aorta and arch. Management of the aortic root depended upon its size, morphology and function, and involvement of the aortic valve.^19^

### Clinical sample

In all, 30 patients admitted to the emergency department of the Ningde Municipal Hospital of Ningde Normal University who met the diagnostic criteria of TAAD were collected. Aortic wall tissues of ascending aorta of patients with TAAD were obtained during surgical operations and the aortic tissues of ascending aorta from donors for heart transplants were selected as the control group. Patients in the control group were excluded from Marfan syndrome, Ehlers-Danlos syndrome, familial thoracic abdominal aortic dissection, hyperkinetic inflammation, and other aortic diseases. A detailed description of the clinical characteristics of the study population is presented in Table 1. This study was approved by the Medical Ethics Committee of Ningde Municipal Hospital of Ningde Normal University (n° 201906F23). All subjects received informed consent. Aortic tissue was fixed with neutral formalin and stored at −80 °C in EP tubes for further analysis.

**Table 1:** The clinical characteristics of TAAD and control groups.

Characteristics	Control (*n* = 30)	TAAD (*n* = 30)
Age (years)	53.2 ± 9.6	52.4 ± 11.5
Body mass index	26.4 ± 4.0	28.5 ± 3.1
Sex		
Male	25	24
Female	5	6
Current smoker	7	8
Alcoholic abuse	0	2
Hypertension	0	26
Hyperlipidemia	0	0
Atherosclerosis	6	15
Diabetes mellitus	0	0

TAAD, Type A Aortic Dissection.

### Cell culture

Rat aorta VSMCs (Chinese Academy of Sciences) were cultured at 37 °C and 5 % CO_2_ in DMEM medium (Invitrogen) containing 10 % FBS (Invitrogen) and necessary antibiotics.

### Cell transfection

Specific oligonucleotides and plasmids were designed, including small interfering RNA (siRNA) targeting HCG18 (si-HCG18), miR-103a-3p mimic, HMGA2-overexpression vector (oe-HMGA2), and negative controls (Sangon, Shanghai, China). Lentiviruses were constructed and packaged by GenePharma (Shanghai, China). Lipofectamine 2000 (Invitrogen) was applied for cell transfection as per the manufacturer's instructions. VSMCs were collected after 48 h of transfection.

### CCK-8 test

Cell viability was assayed by CCK-8. Specifically. VSMCs were re-suspended in DMEM and plated on 96-well plates (1 × 10^4^ cells/well). Then, the CCK-8 solution (10 μL/well; Dojindo) was incubated at 37 °C for 2 h before recording the absorbance at 450 nm on the microplate reader (Thermo Fisher Scientific).

### Colony formation experiment

Cell proliferation was detected by colony formation assay. Specifically, VSMCs were inoculated in 6-well plates (700-well) for 14d After being fixed in 10 % formaldehyde, VSMCs were stained with 1 % crystal violet (Beyotime, China) and imaged under a microscope (Leica, Germany) to count colonies (≥ 50 cells).

### Flow cytometry

Apoptosis was detected by flow cytometry. Specifically, VSMCs were digested with trypsin, followed by staining with Annexin V-FITC/PI Apoptosis Detection Kit (BD Biosciences) and sorting on the FACScan machine (BD Biosciences). Cell apoptosis rate was determined by CellQuest software (BD Biosciences).

### Luciferase activity measurement

Wild Type (WT)-HCG18, Mutant (MUT)-HCG18, WT-HMGA2, and MUT-HMGA2 vectors were prepared by Sangon. The vectors and miR-103a-3p mimic or mimic NC and were co-transfected into VSMCs. Analysis of luciferase activity was made using the dual luciferase reporting kit (Promega) and GloMax fluorescence reader (Promega).

### Rat model of AD

β-Aminopropionitrile (BAPN) can inhibit the crosslinking of collagen and elastin fibers, leading to AD.^20^^,^^21^ AD animal models were established^[^^22^^,^^23^ as described previously. AD rats (3-weeks of age) were fed a normal diet with 0.25 % BAPN (Sigma) orally until 7-weeks of age. The sham rats were treated with the same amount of normal saline. Then, at 6-weeks of age, AD rats have injected with either si-HCG18 or Negative Controls (si-NC) lentivirus (1 × 10^11^ PFU) through the caudal vein. Finally, the aorta tissues were collected from each euthanized rat and fixed in 4 % paraformaldehyde (P0099, Beyotime). Animal experiments complied with the ARRIVE guidelines and performed in accordance with the National Institutes of Health Guide for the Care and Use of Laboratory Animals. The experiments were approved by the Institutional Animal Care and Use Committee of Ningde Municipal Hospital of Ningde Normal University (n° 201908J11).

### HE-staining

The fixed aorta tissue was embedded in paraffin and sectioned into 4 μm. Tissue sections were dewaxed in xylene and rehydrated in graded ethanol. Staining experiments were performed using the HE-kit (C0105, Beyotime). After the sections were dehydrated, cleared, and sealed, the histopathological changes in the aortic tissue were observed under a microscope (Olympus, Japan).

### RT-qPCR

Total RNA was isolated using TRIzol reagent (Invitrogen) and then reverse-transcribed into cDNA using the PrimeScript RT Master Mix Kit (Takara). Real-time qPCR analysis was performed using SYBR Green Realtime PCR Master Mix (ToyoBo) in the 7900HT PCR System (ABI). All primers (Table 2) for RT-qPCR were synthesized by Sangon. GAPDH and U6 were considered internal controls for lncRNA/mRNA and miRNA analysis, respectively.

**Table 2:** RT-qPCR sequences.

**Genes**	**Sequences (5′–3′)**

HCG18	Forward: GCTAGGTCCTCTACTTTCTG
	Reverse: CAGAAAGTAGAGGACCTAGC
miR-103a-3p	Forward: AGCAGCATTGTACAGGGCTATG
	Reverse: CTCTACAGCTATATTGCCAGCCAC
HMGA2	Forward: GGGCGCCGACATTCAAT
	Reverse: ACTGCAGTGTCTTCTCCCTTCAA
U6	Forward: CTCGCTTCGGCAGCACA
	Reverse: AACGCTTCACGAATTTGCGT
GAPDH	Forward: GTCGGTGTGAACGGATTTG
	Reverse: TCCCATTCTCAGCCTTGAC

HCG18, Long Noncoding RNA HLA Complex Group 18; miR-103a-3p, microRNA-103a-3p; HMGA2, High Mobility Group AT-hook 2; GAPDH, Glyceraldehyde-3-Phosphate Dehydrogenase.

### Western blot

After extracting total proteins from the aortic tissue and VSMCs using RIPA lysis buffer, protein concentration was determined using the BCA kit. Protein (20 μg) was separated using 10 % SDS-PAGE, loaded onto the PVDF membrane (Merk, Germany), and sealed with 5 % skim milk for 2 h before mixing with primary antibody HMGA2 (ab97276, 1:1000, Abcam), Bcl-2 (SC-7382, 1:500, Santa Cruz), Bax (SC-7480, 1: 500, Santa Cruz), and GAPDH (ab8245, 1:1000, Abcam) overnight at 4 °C and corresponding secondary antibody for 2 h. Protein bands were visualized using ECL reagents (Pierce) and imaged using FluorChem imaging systems (BioRad).

### Statistical analysis

All data were processed with GraphPad Prism 6.0 software. Values were expressed as mean ± standard deviation and assessed by *t*-test (two groups) or one-way ANOVA (multiple groups); *p* < 0.05 indicated statistical significance.

## Results

### Downregulating HCG18 enhances VSMC proliferation and reduces apoptosis

**Fig. 1:**
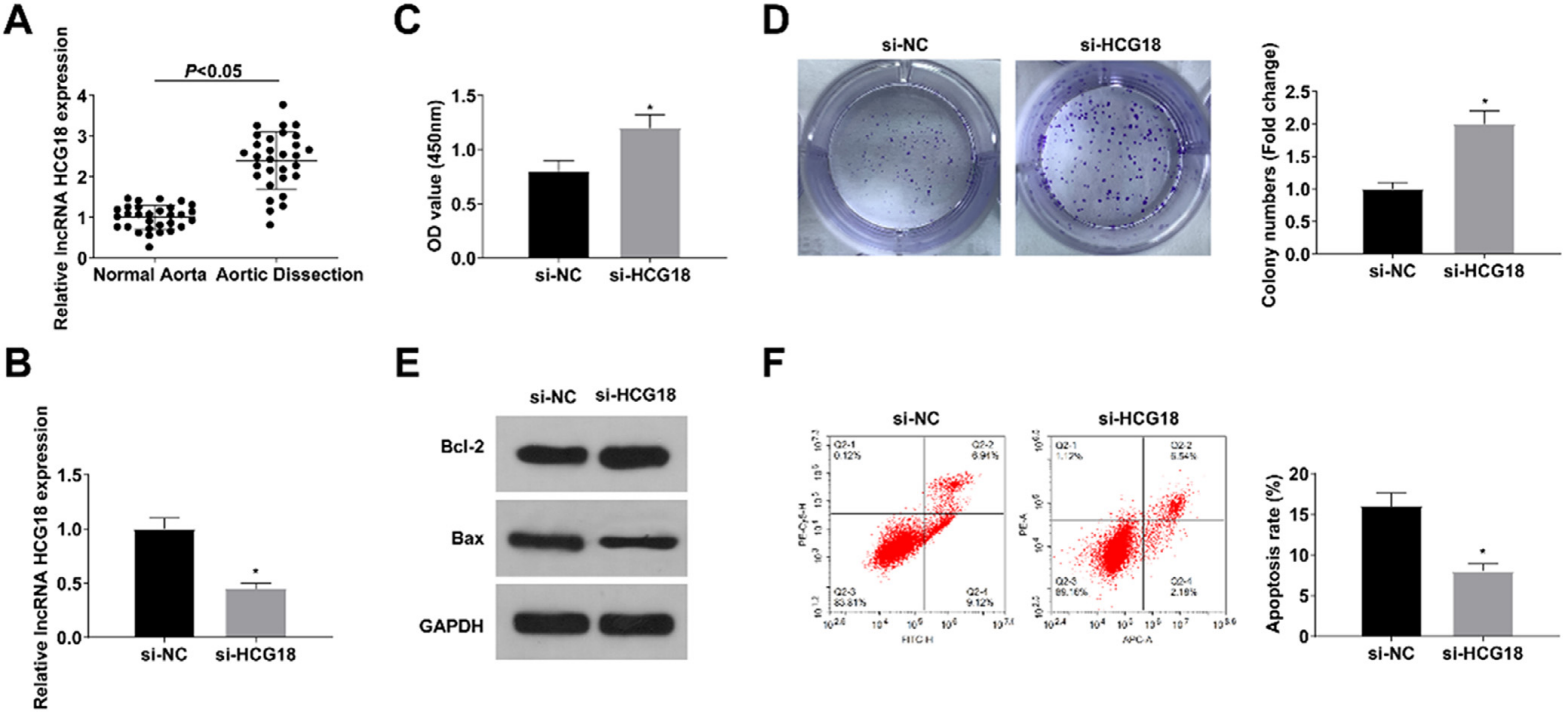
Downregulating HCG18 promotes the proliferation of VSMCs and inhibits cell apoptosis. (A) RT-qPCR analyzed HCG18 in aortic tissues of AD patients (*n* = 30); (B) RT-qPCR verified the successful transfection; (C‒D) CCK-8 and colony formation assay measured VSMC proliferation; (E) Western blot measured Bcl-2 and Bax protein expression; (F) Flow cytometry analyzed VSMC apoptosis. Values are expressed as mean ± standard deviation (*n* = 3). *vs*.* si-NC, *p* < 0.05.

In the aortic tissues of TAAD patients, HCG18 levels were increased (*p* < 0.05; Fig. 1A). To explore HCG18’s role in AD, HCG18’s effect on the proliferation and apoptosis of VSMCs was examined. si-NC or si-HCG18 was transfected into VSMCs, and RT-qPCR results revealed that HCG18 expression was reduced in VSMCs transfected with si-HCG18 (*p* < 0.05; Fig. 1B). CCK-8 and colony formation assays showed that silencing HCG18 promoted the proliferation of VSMCs (*p* < 0.05; Fig. 1C‒D). Western blot results revealed that down-regulation of HCG18 increased Bcl-2 protein levels and decreased Bax protein levels (*p* < 0.05; Fig. 1E). Flow cytometry results showed that down-regulation of HCG18 inhibited apoptosis in VSMCs (*p* < 0.05; Fig. 1F).

### HCG18 acts as a miR-103a-3p sponge

**Fig. 2:**
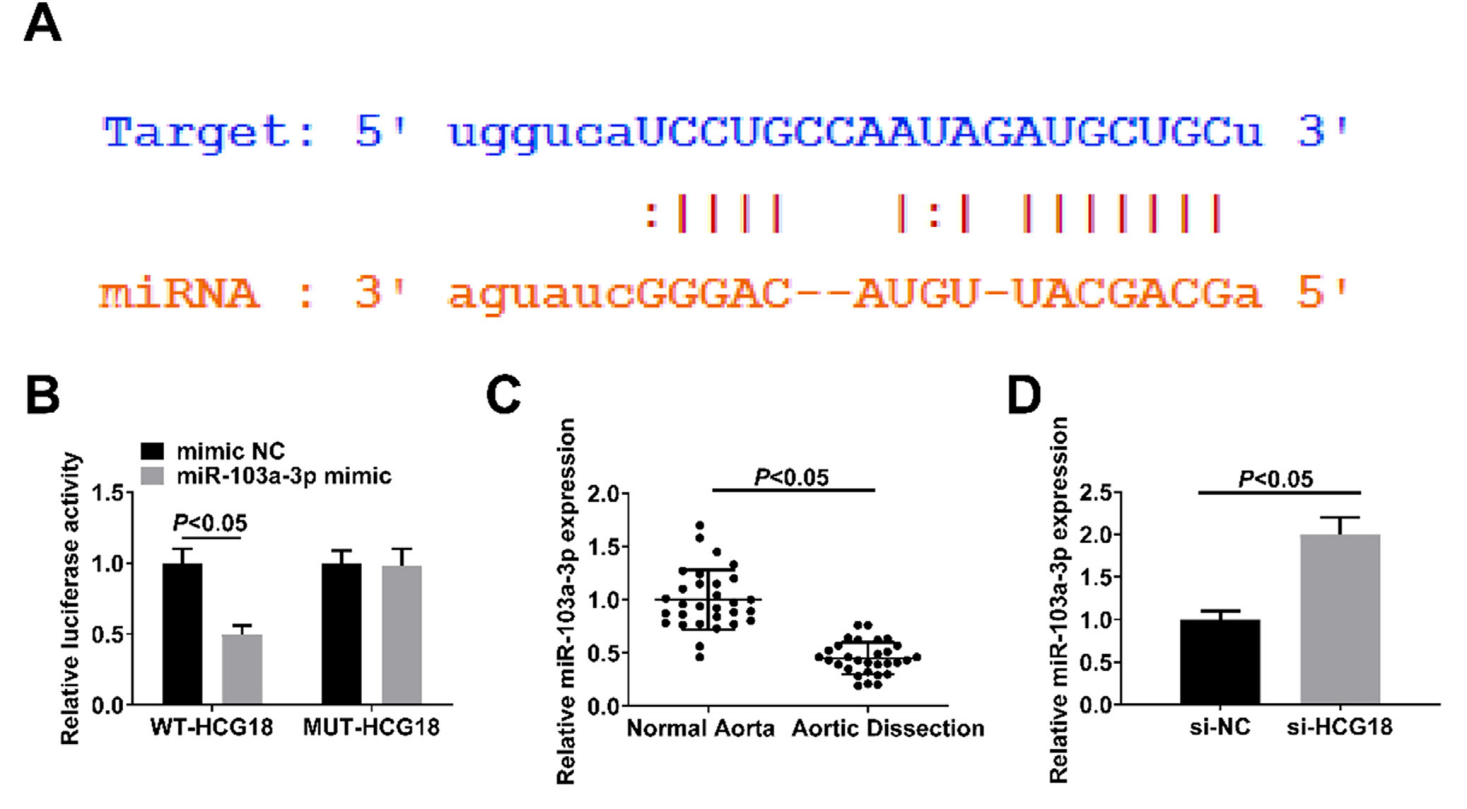
HCG18 functions as a miR-103a-3p sponge. (A) Bioinformation website predicted the binding sites of HCG18 and miR-103a-3p; (B) Dual luciferase reporter assay verified the targeting relationship between HCG18 and miR-103a-3p; (C) RT-qPCR analyzed miR-103a-3p expression in aortic tissues of AD patients (*n* = 30); (D) RT-qPCR analyzed miR-103a-3p expression in VSMCs transfected with si-NC or si-HCG18. Values are expressed as mean ± standard deviation (*n* = 3).

Binding sites between HCG18 and miR-103a-3p were found through bioinformation website analysis (Fig. 2A). Then, dual luciferase reporter assay verified the targeting relationship between HCG18 and miR-103a-3p, as manifested by the decrease in luciferase activity after co-transfection of WT-HCG18 with miR-103a-3p mimic (*p* < 0.05; Fig. 2B). RT-qPCR results revealed downregulated miR-103a-3p in the aortic tissues of TAAD patients (*p* < 0.05; Fig. 2C), while increased expression in si-HCG18-transfected VSMCs (*p* < 0.05; Fig. 2D).

### Upregulating miR-103a-3p protects VSMCs

**Fig. 3:**
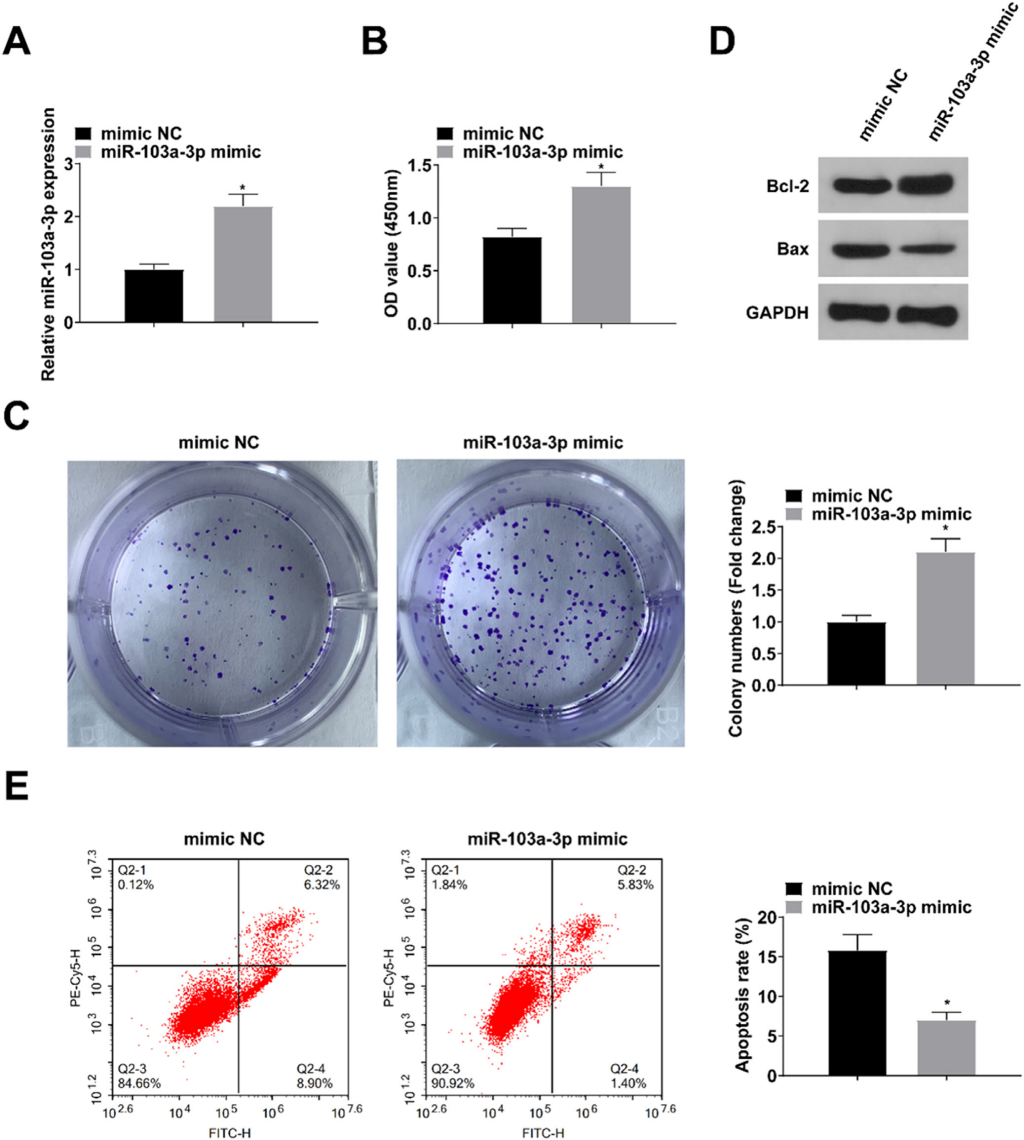
Upregulating miR-103a-3p protects VSMCs. (A) RT-qPCR verified the successful transfection; (B‒C) CCK-8 and colony formation assay measured VSMC proliferation; (D) Western blot measurement measured Bcl-2 and Bax protein expression; (E) Flow cytometry analyzed VSMC apoptosis. Values are expressed as mean ± standard deviation (*n* = 3). *vs. mimic NC, *p* < 0.05.

mimic NC or miR-103a-3p mimic was transfected into VSMCs. RT-qPCR assay showed elevated miR-103a-3p expression in VSMCs transfected with miR-103a-3p mimic (*p* < 0.05; Fig. 3A). CCK-8 and colony formation assays showed that upregulation of miR-103a-3p promoted the proliferation of VSMCs (both *p* < 0.05; Fig. 3B‒C). Western blot results revealed that up-regulation of miR-103a-3p resulted in increased levels of Bcl-2 protein and decreased levels of Bax protein (*p* < 0.05; Fig. 3D). Flow cytometry results showed that up-regulation of miR-103a-3p inhibited apoptosis in VSMCs (*p* < 0.05; Fig. 3E).

### miR-103a-3p targets HMGA2

**Fig. 4:**
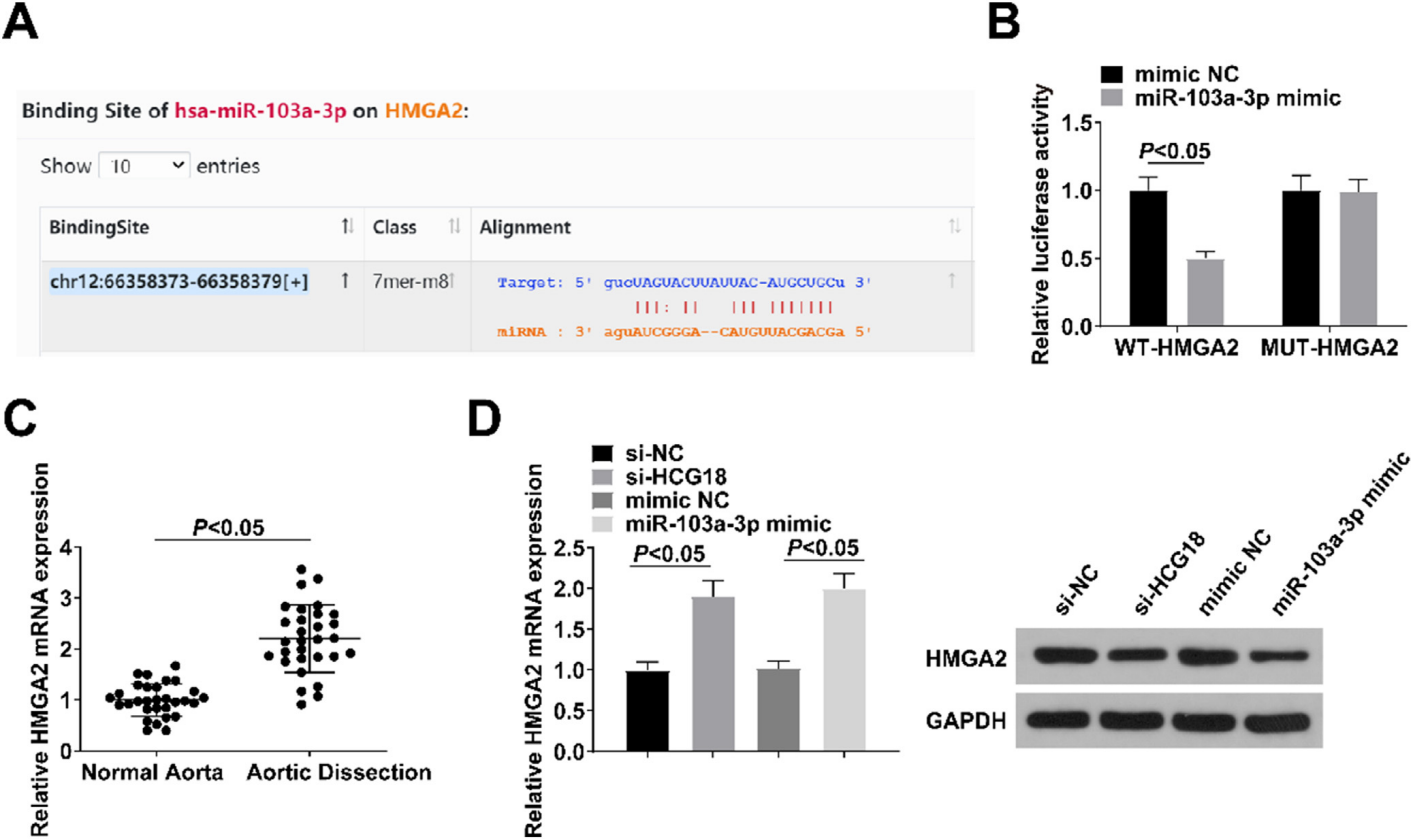
HMGA2 is directly targeted by miR-103a-3p. (A) Bioinformation website predicted the binding sites of miR-103a-3p to HMGA2; (B) Dual luciferase reporter assay verified the targeting relationship between miR-103a-3p and HMGA2; (C) RT-qPCR analyzed HMGA2 in aortic tissues of AD patients (*n* = 30); (D) RT-qPCR and Western blot measurement analyzed HMGA2 in VSMCs transfected with si-NC, si-HCG18, mimic NC, or miR-103a-3p mimic. Values are expressed as mean ± standard deviation (*n* = 3).

Bioinformatics website analysis showed that miR-103a-3p has a targeted binding site with HMGA2 (Fig. 4A). The results of dual luciferase reporter gene assay experiments showed that the luciferase activity was reduced after WT-HMGA2 was co-transfected with miR-103a-3p mimic (*p* < 0.05; Fig. 4B). HMGA2 was upregulated in the aortic tissues of patients with TAAD (*p* < 0.05; Fig. 4C) and was decreased in VSMCs transfected with si-HCG18 or miR-103a-3p mimic (*p* < 0.05; Fig. 4D).

### HCG18/miR-103a-3p/HMGA2 axis affects VSMC proliferation and apoptosis

**Fig. 5:**
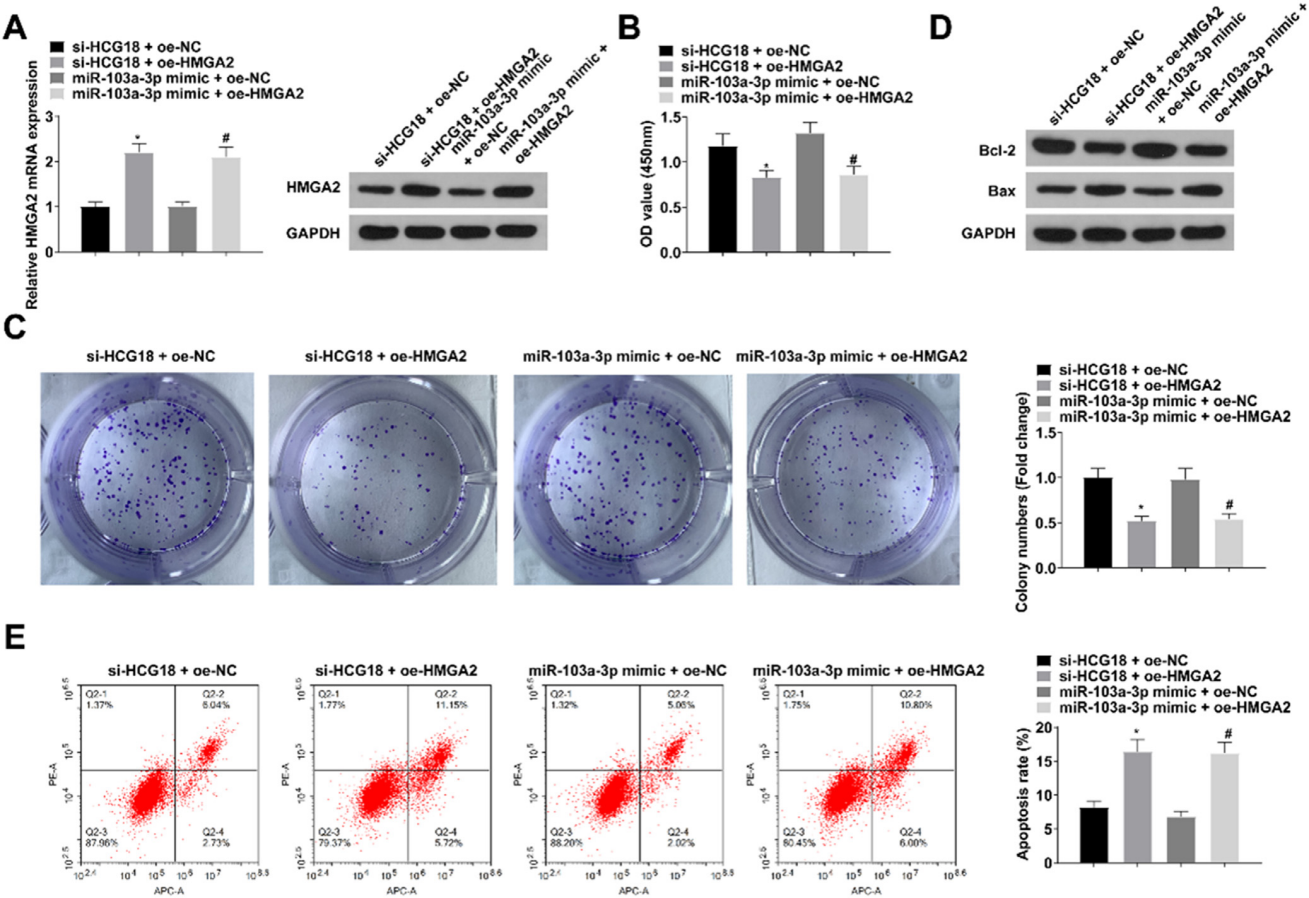
HCG18/miR-103a-3p/HMGA2 axis affects VSMC proliferation and apoptosis. (A) RT-qPCR and Western blot measurement verified the successful transfection; (B‒C) CCK-8 and colony formation assay measured VSMC proliferation; (D) Western blot measurement measured Bcl-2 and Bax protein expression; (E) Flow cytometry analyzed VSMC apoptosis. Values are expressed as mean ± standard deviation (*n* = 3). *vs. si-HCG18 + oe-NC, *p* < 0.05; ^#^vs. miR-103a-3p mimic + oe-NC, *p* < 0.05.

To further study HCG18/miR-103a-3p/HMGA2 axis in AD, si-HCG18 + oe-NC, si-HCG18 + oe-HMGA2, miR-103a-3p mimic + oe-NC or miR-103a-3p mimic + oe-HMGA2 were transfected into VSMCs. As measured by RT-qPCR and Western blot measurement, oe-HMGA2 impaired si-HCG18 or miR-103a-3p mimic in reducing HCG18 levels (Fig. 5A). Experimental data measured that enhancing HMGA2 could reduce the impacts of HCG18 downregulation or miR-103a-3p upregulation on proliferation and apoptosis of VSMCs (all *p* < 0.05; Fig. 5B‒E).

### Downregulating HCG18 improves the pathological injury of the aorta in AD rats

**Fig. 6:**
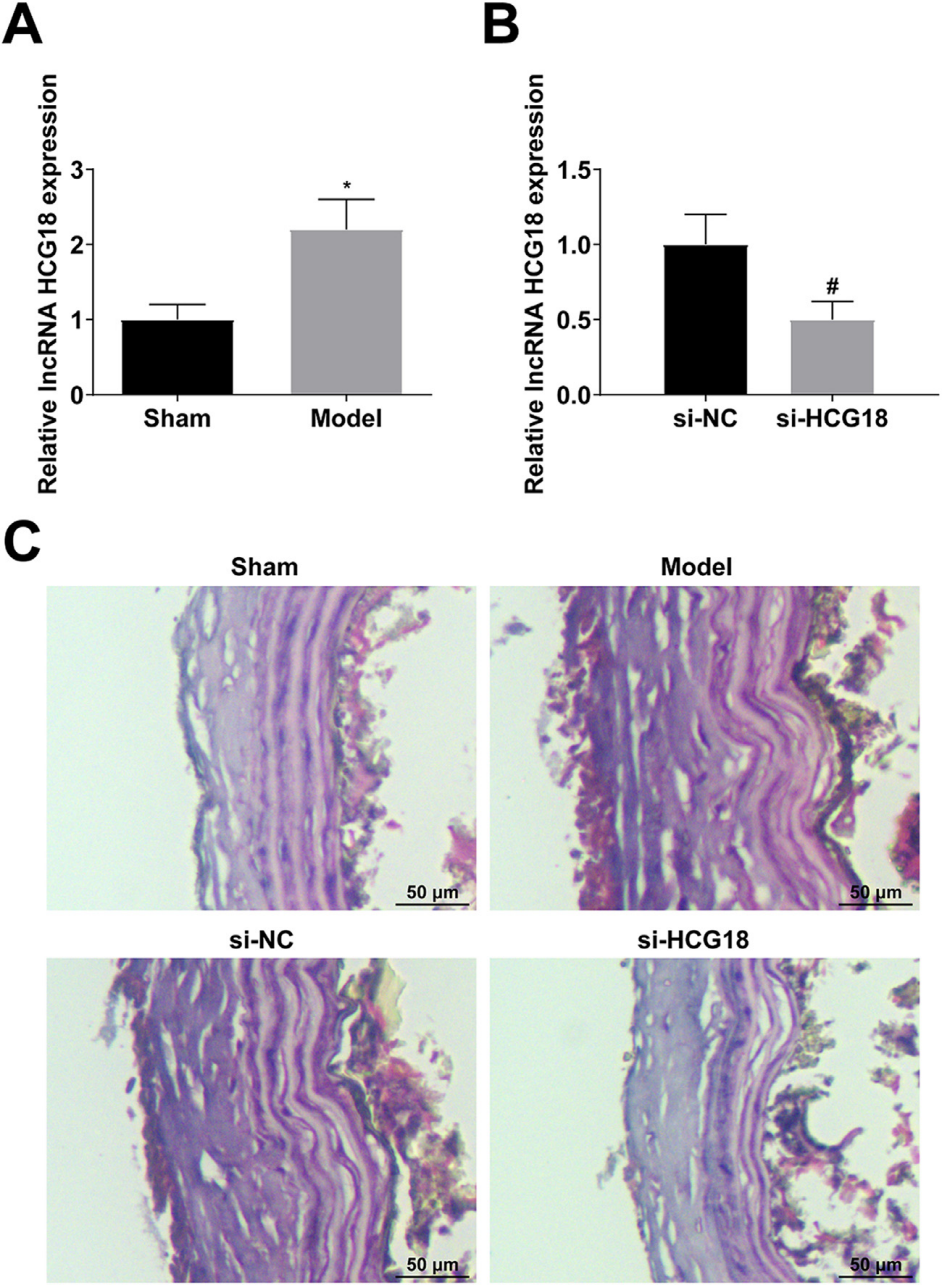
Downregulating HCG18 improves the pathological injury of aorta in AD rats. (A) RT-qPCR analyzed HCG18 in AD rats; (B) RT-qPCR verified the successful injection of lentivirus; (C) HE-staining observed the pathological changes of aorta in AD rats; Values are expressed as mean ± standard deviation (*n* = 6). *vs. Sham, *p* < 0.05; ^#^vs. si-NC, *p* < 0.05.

Finally, an AD animal model was induced to verify the regulatory effect of HCG18. HCG18 was upregulated in AD rats (*p* < 0.05; Fig. 6A). si-NC or si-HCG18 lentivirus was injected into the tail vein of AD rats, and successful lentivirus injection was verified by RT-qPCR (*p* < 0.05; Fig. 6B). HE-staining observed that the aortic wall of AD rats contained broken elastic fibers, and blood cells entered the aortic wall, leading to tearing and stripping; down-regulation of HCG18 could improve the above pathological changes (Fig. 6C).

## Discussion

It is known that HCG18 exerts adjustive effects on VSMCs.^16^ This work studied the function of HCG18 in AD and explored its related downstream mechanism. Abnormal regulation of lncRNAs is often related to pathological processes, including coronary heart disease.^24^^,^^25^ LncRNAs have also been reported to be involved in regulating cardiovascular diseases such as heart failure, hypertension, and aneurysm.^26^^,^^27^ HCG18 is a recently studied lncRNA that is abnormally expressed in many diseases.^28^, ^29^, ^30^ The current work identified that HCG18 was upregulated in the aortic tissues of TAAD patients, and down-regulated HCG18 protected VSMCs regarding cell proliferation and apoptosis. In addition, down-regulated HCG18 could improve the pathological injury of aorta in AD rats.

Studies have confirmed the mutual regulatory relationship between lncRNAs and miRNAs, exerting physiological and pathological functions through different regulatory ways.^31^, ^32^, ^33^ It is noted that HCG18 aggravates diabetic peripheral neuropathy through modulating miR-146a.^34^ It has been proposed that HCG18 accelerates lung adenocarcinoma development by targeting miR-34a-5p.^35^ The present study found that HCG18 was competitively bound to miR-103a-3p. As measured, miR-103a-3p was downregulated in the aortic tissues of AD patients and enhancing miR-103a-3p induced proliferative and suppressed apoptotic activities of VSMCs.

HMGA2, as a transcription factor, is increased in expression in malignant tumors.^36^, ^37^, ^38^ As suggested, targeting HMGA2 is a useful method in miR-26b inhibiting Stanford Type A AD.^39^ The current work identified HMGA2 as the target gene of miR-103a-3p, and HMGA2 was upregulated in the aortic tissues of AD patients. HMGA2 promotion could impair HCG18 downregulation or miR-103a-3p upregulation in mediating VSMC proliferation and apoptosis.

Considering the limitations of this study, a limited sample may not fully confirm the accuracy of the results. Secondly, the relationship between HCG18 and other potential targeted miRNAs needs further attention and research. In addition, only TAAD patients were included in this study, and the experimental results cannot be generalized to all AD patients for the time being.

## Conclusion

In summary, this study for the first time found that HCG18 promotes AD progression by competing with miR-103a-3p to target HMGA2 expression. This provides a theoretical basis for gene therapy for aortic coarctation, and surgical treatment assisted by gene therapy may be a new therapeutic strategy for patients with TAAD.

## Availability of data and materials

The datasets used and/or analyzed during the present study are available from the corresponding author upon reasonable request.

## Ethics statement

This study was approved by the Medical Ethics Committee of Ningde Municipal Hospital of Ningde Normal University (n°201906F23). All subjects received informed consent. The animal experiments were complied with the ARRIVE guidelines and approved by the ethics committee of Ningde Municipal Hospital of Ningde Normal University (n° 201908J11).

## Authors’ contributions

ZhiHong Yang designed the research study. YuanSheng Cui and ShuGuo Xu performed the research. LongBiao Li provided help and advice. YuanSheng Cui and ShuGuo Xu analyzed the data. ZhiHong Yang wrote the manuscript. LongBiao Li reviewed and edited the manuscript. All authors contributed to editorial changes in the manuscript. All authors read and approved the final manuscript.

## Funding

Not applicable.

## Declaration of competing interest

The authors declare no conflicts of interest.
